# Sex differences in subclinical hypothyroidism and associations with metabolic risk factors: a health examination-based study in mainland China

**DOI:** 10.1186/s12902-020-00586-5

**Published:** 2020-07-06

**Authors:** Li Jiang, Jinman Du, Weizhu Wu, Jianjiang Fang, Jufang Wang, Jinhua Ding

**Affiliations:** 1 Department of Emergency, Ningbo Medical Center Lihuili Hospital, Taipei Medical University Ningbo Medical Center, Ningbo, 315000 China; 2 Health Examination Center, Ningbo Medical Center Lihuili Hospital, Taipei Medical University Ningbo Medical Center, Ningbo, 315000 China; 3 Department of Breast and Thyroid Surgery, Ningbo Medical Center Lihuili Hospital, Taipei Medical University Ningbo Medical Center, Ningbo, 315000 China

**Keywords:** Subclinical hypothyroidism, Metabolic syndrome, Risk factor

## Abstract

**Background:**

The association between subclinical hypothyroidism (SCH) and metabolic risk factors in the general health examination-based population has been widely explored. However, the results have been inconclusive. Additionally, the sex differences in the prevalence of SCH and the association of SCH with metabolic risk factors remain unknown.

**Methods:**

We conducted this cross-sectional study using data from health examination-based participants between June 2016 and April 2018 in our health examination centre. Sex differences SCH and the association of SCH with metabolic risk factors were explored.

**Results:**

The total prevalence of SCH was 3.40% among the 5319 included participants, and 4.90% among the 2306 female participants, which was much higher than the prevalence of 2.26% among the 3013 male participants (*p* < 0.05). In males, the difference between participants younger than 60 and aged 60 or older was not significant (*p* = 0.104); while in females, the difference between participants younger than 40 and participants aged 40 or older was statistically significant (*p* = 0.023). Multivariate logistic regression analysis demonstrated that age (OR = 0.568, *p* = 0.004), body-mass index (BMI) (OR = 5.029, *p* < 0.001) and systolic/diastolic blood pressure (SBP/DBP) (OR = 5.243, *p* < 0.001) were independent predictors of SCH in females, but no metabolic risk factor was significantly associated with SCH in males. Further analysis revealed that the prevalence was much higher in participants with one or two metabolic risk factors than in those with no above metabolic risk factors regardless of age (*p* < 0.01).

**Conclusions:**

Our study demonstrates that high BMI and/or high blood pressure are associated with SCH in female participants, and the prevalence of SCH among women with one or two metabolic risk factors ranges from 7.69–14.81%, which indicates that in such a population, serum concentrations of TSH and FT4 may be routinely screened in mainland China. Certainly, prospective, large-scale studies with long follow-up period are still necessary to further verify our results.

## Background

Subclinical hypothyroidism (SCH) is a mild thyroid disorder with elevated thyroid-stimulating hormone (TSH) concentration and normal concentration of serum free thyroxin. Numerous studies demonstrate that SCH is associated with cardiac disease [1], higher low-density lipoprotein cholesterol [2], and depression and cognitive dysfunction [3], which together result in an increased risk for cardiovascular disease (CVD)-related death [4]. It is reported that the prevalence ranges substantially from 4 to 20% [5–9], and such a wide range of prevalence is closely related to the reference range of TSH and the population characteristics, and other factors such as sex, area of residence, iodine intake and some auto-antibodies also have great influence on the prevalence [8–10]. On the one hand, the presence of SCH can result in the increasing rate of CVD [4]; On the other hand, SCH is usually clinically asymptomatic and accidentally due to the presence of non-specific symptoms [11]. Therefore, it is necessary to screen this potential disease by serum TSH test. When cost-effectiveness is considered, due to the relatively low prevalence of SCH in the general population, thus TSH screening is not routinely recommended by the leading association of thyroid medicine [12–15]. However, in certain population whose risks for SCH is much higher, TSH screening may be an appropriate option, because higher rate of SCH population can be identified, which means more attention should be paid on such population and the risks of CVD may decrease.

Metabolic syndrome (MetS) is not a single disease, but a cluster of metabolic risk factors which include visceral obesity, hypertension, hyperglycemia, dyslipidaemia, and atherogenisis [16]. The associations between MetS and SCH have been widely explored with inconsistent results. SCH is closely associated with MetS in several previous studies [17–19], however, another study does not find definite association between them [20]. Additionally, it is reported that SCH is also associated with partial components of MetS, but not all components [19, 20]. It is considered that some factors including age, sex, and body-mass index (BMI) may contribute to these conflicting results.

A cross-sectional study reported the prevalence of SCH in 1150 university employees in mainland China [21]; however, the small sample size was an obvious defect. Additionally, whether there is any difference in SCH by sex, and whether there is a certain population whose risk for SCH is significantly higher than that in the general population remain unexplored. Therefore, our study in a large-scale Chinese population aims to investigate the prevalence of SCH and sex differences in SCH, to explore the associations between SCH and metabolic risk factors by sex, and to identify a certain population whose risk for SCH is much higher than that of the general population.

## Methods

### Study population

Before data collection, Research Ethics Committee in Ningbo Medical Center Lihuili Hospital has approved the study. Written informed consent for using the screening results for academic research was obtained from every participant. Both doctor Du and doctor Wang were the administrators of health examination center, and had necessary permissions to the database.

**Fig. 1:**
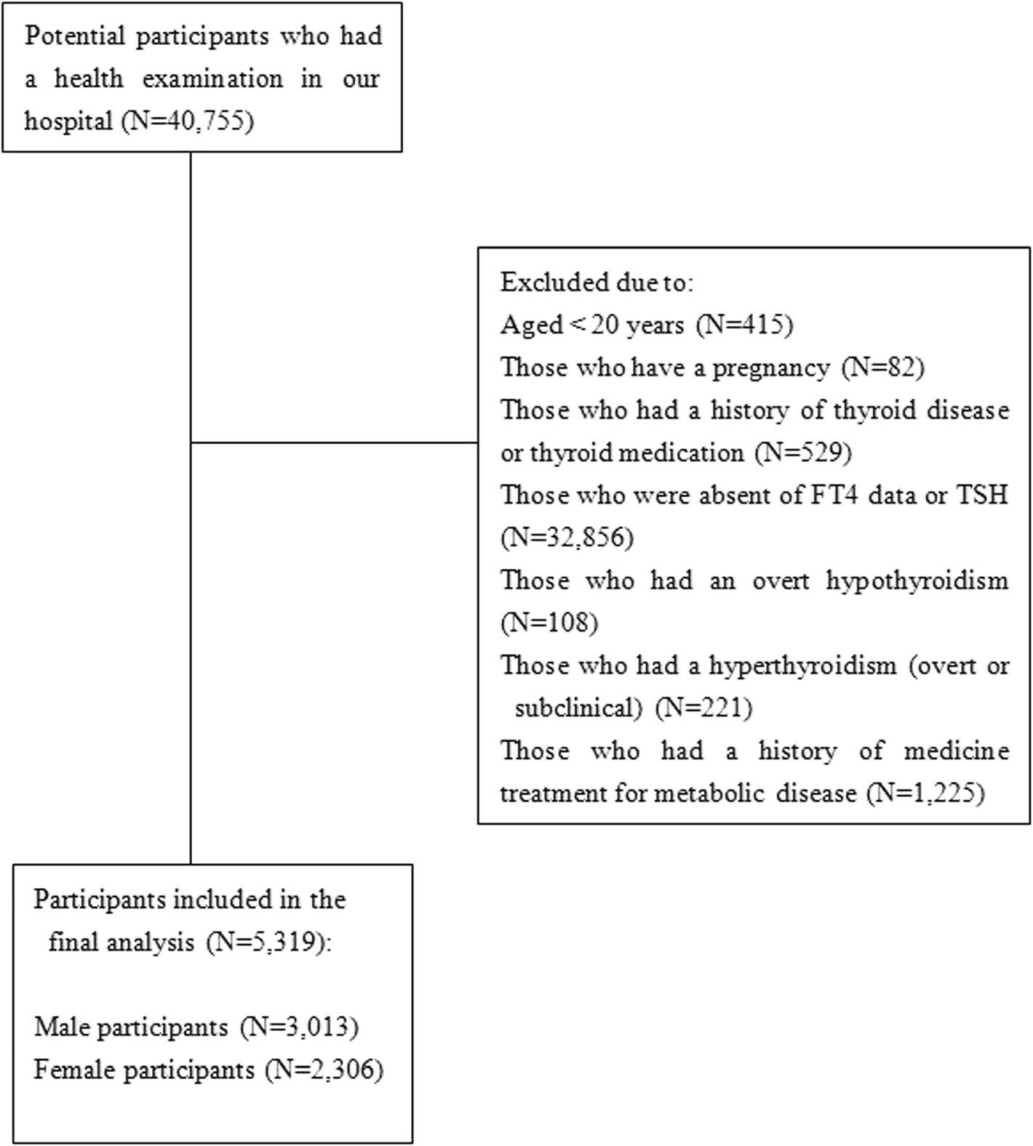
Flow chart of the study

Individuals who had a health examination in the health examination centre of Ningbo Medical Center Lihuili Hospital between June 2016 and April 2018 were potential participants. Participants had a normal occupation, lived in the local area for more than 5 years, and had a regular health examination once a year. Participants in this study had complete data on thyroid function. Exclusion criteria were as the followings: (1) age < 20; (2) pregnancy or within the first year of the postpartum period; (3) subclinical or overt hyperthyroidism (serum TSH > 0.35 mIU/L with elevated free tetraiodothyronine (FT4)) or overt hypothyroidism (serum TSH < 4.94 mIU/L with low FT4); (4) a history of thyroid disease; (5) a clear history of metabolic diseases such as hyperlipidaemia, hypertension, hyperglycaemia, hyperuricemia and concurrent with medication for these disease; and (6) insufficient data. Figure 1 shows the flow chart of the included participants.

### Data collection

The following data were collected from the health examination database in our hospital: sex, age, height, weight, BMI, fasting blood glucose (AC), high/low-density lipoprotein cholesterol (HDL-C/LDL-C), triglycerides (TGs), serum creatinine (Scr), uric acid (UA), FT3, FT4 and TSH. Anthropometric measurements of height (cm) and weight (kg) were taken using a height and weight machine while participants were barefoot and wearing light clothes. BMI was calculated from the measured height and weight. Blood pressure (mmHg) was measured with the participants in a seated position after 10 min of rest using an electronic sphygmomanometer. Biochemical parameters including AC, TGs, HDL-C, LDL-C, UA, Scr, TSH, FT3 and FT4 were tested from fasting blood samples.

### Definition of SCH and metabolic risk factors

The definition of SCH based on a normal serum FT4 concentration with an elevated serum TSH concentration has been widely used in previous literature. However, the reference range used for TSH has been inconsistent, and the upper limit has varied in previous literature. In our clinical practice, TSH was measured using an E-TSH kit (Roche Diagnostics), for which the reference range was 0.35–5.00 μIU/mL. Therefore, in our study, the concentration of TSH > 5.0 μIU/mL was considered to exceed the upper limit. The concentration > 10.0 μIU/mL often indicated overt hypothyroidism and was often accompanied by alterations in lipid and carbohydrate metabolism. Therefore, the concentration of TSH within the range from 5.0 to 10.0 μIU/mL, and with a normal FT4 concentration were used to define SCH in our study.

The definition of metabolic risk factors was adopted as well-accepted criteria. Either or both of the blood pressures, systolic blood pressure (SBP) > 130 mmHg and diastolic blood pressure (DBP) > 85 mmHg, were categorized as hypertension; and a concentration of TG > 150 mg/dL was categorized as hypertriglyceridemia. Obesity was defined as BMI ≥ 25Kg/m2; hyperglycemia was defined as a concentration of AC > 100 mg/dL; and low HDL was defined as a concentration of HDL < 50 mg/dL for men and 40 mg/dL for women.

### Statistical analysis

Participants were categorized into two groups based on the concentration of TSH: the euthyroid (EUT) group with a normal serum FT4 concentration and a TSH concentration between 0.35 and 5.0 μIU/mL; and the SCH group with a normal serum FT4 concentration and a TSH concentration between 5.0 and 10.0 μIU/mL (the participants with TSH > 10.0 μIU/mL were not included in the current study). Between-group comparisons (univariate analysis adjusted for BMI and age, because they were important confounders for SCH in previous studies) were performed using the χ2 (Chi-square) test for categorical variables and the t-test for continuous variables (mean ± standard deviation, M ± SD). Then, multivariate logistic regression analysis adjusting for variables from the univariate analysis that were associated with SCH and those well-accepted variables such as age and BMI, was performed to test factors’ independence. In addition, the interaction of independent predictors determined by multivariate logistic regression analysis was also assessed. Statistical analyses were performed using SPSS 19.0 software (SPSS, Chicago, IL, http://www.spss.com). Statistical test was two-sided. *P* value less than 0.05 was considered statistically significant, and the odds ratio (OR) as well as 95% confidence interval (CI) were also calculated.

## Results

### Demographic characteristics and clinical data of participants by sex

A total of 5319 participants visiting the health examination centre of Ningbo Medical Center Lihuili Hospital were included in this study. The mean age was 42.5 ± 11.7 years, ranging from 20 to 82 years, and 3013 participants (56.97%) were men. Table 1 demonstrates the demographic characteristics and clinical data of all participants and participants by sex.

**Table 1:** Participants’ demographic and clinical characteristics in overall participants and by sex

Variables	Overall (*n* = 5319)	Male (*n* = 3013)	Female (*n* = 2306)	*p*
Age (yrs)	42.53 ± 11.7	43.24 ± 11.40	41.62 ± 11.91	< 0.001
Education (yrs)	10.5 ± 3.81	10.8 ± 3.72	10.2 ± 3.90	< 0.001
Height (cm)	166.6 ± 8.0	171.60 ± 6.02	160.24 ± 6.30	< 0.001
Weight (kg)	65.4 ± 12.32	71.99 ± 10.65	56.37 ± 8.26	< 0.001
BMI^a^ (kg/m ^**2**^)	23.4 ± 3.3	24.50 ± 3.26	21.93 ± 2.98	< 0.001
Waist circumference	82.62 ± 6.47	87.52 ± 5.18	77.23 ± 5.05	< 0.001
SBP (mm Hg)	120.9 ± 16.9	123.91 ± 16.27	116.74 ± 16.74	< 0.001
DBP (mm Hg)	74.2 ± 11.4	77.12 ± 11.43	70.72 ± 10.51	< 0.001
AC (mg/dL)	5.3 ± 1.1	5.35 ± 1.11	5.26 ± 1.03	< 0.001
Total Chol (mg/dL)	185.6 ± 34.8	187.53 ± 35.22	183.95 ± 35.18	< 0.001
TG (mg/dL)	136.6 ± 123.87	160.27 ± 143.56	104.79 ± 74.17	< 0.001
HDL-C (mg/dL)	54.43 ± 14.10	50.07 ± 11.22	60.94 ± 15.06	< 0.001
LDL-C (mg/dL)	92.6 ± 23.6	86.42 ± 19.52	98.05 ± 27.13	< 0.001
UA (mg/dL)	356.0 ± 91.3	400.01 ± 77.16	282.12 ± 60.23	< 0.001
Scr (umol/L)	69.18 ± 14.38	77.31 ± 10.70	55.56 ± 8.06	< 0.001
TSH concentration (mIU/L)	2.13 ± 2.51	1.97 ± 2.32	2.30 ± 2.72	< 0.001
Free-T_3_ concentration (pmol/L)	5.07 ± 0.55	5.29 ± 0.51	4.79 ± 0.48	< 0.001
Free-T4 concentration (pmol/L)	16.23 ± 1.95	16.71 ± 1.94	15.60 ± 1.77	< 0.001

Data were presented as mean ± SD. Groups were compared using t-test.

^a^BMI: calculated as weight (in kilogram) divided by height (in meter) squared

*Abbreviation*: *BMI* body mass index, *SBP* systolic blood pressure, *DBP* diastolic blood pressure, *WBC* white blood cell, *RBC* red blood cell, *PLT* platelets; hemoglobin, *hs-CRP* high sensitivity C-reactive protein, *AC* fasting blood glucose, *Total Chol* cholesterol, *TG* triglyceride, *HDL-C* high density lipoprotein cholesterol, *LDL-C* low density lipoprotein cholesterol, *UA* uric acid; Scr: serum creatinine, *TSH* Thyroid Stimulating Hormone, *Free-T3* free triiodothyroxine, *Free-T4* free tetraiodothyroxine

*p* < 0.05 was considered as statistically significance

### Prevalence of SCH in different age groups stratified by sex

There were a total of 181 SCH cases in the overall participants, with a prevalence of 3.40% (181/5319). The prevalence of SCH was 4.90% (113/2306) and 2.26% (68/3013) in female and male participants, respectively, and the difference by sex was significant (*p* < 0.001).

**Fig. 2:**
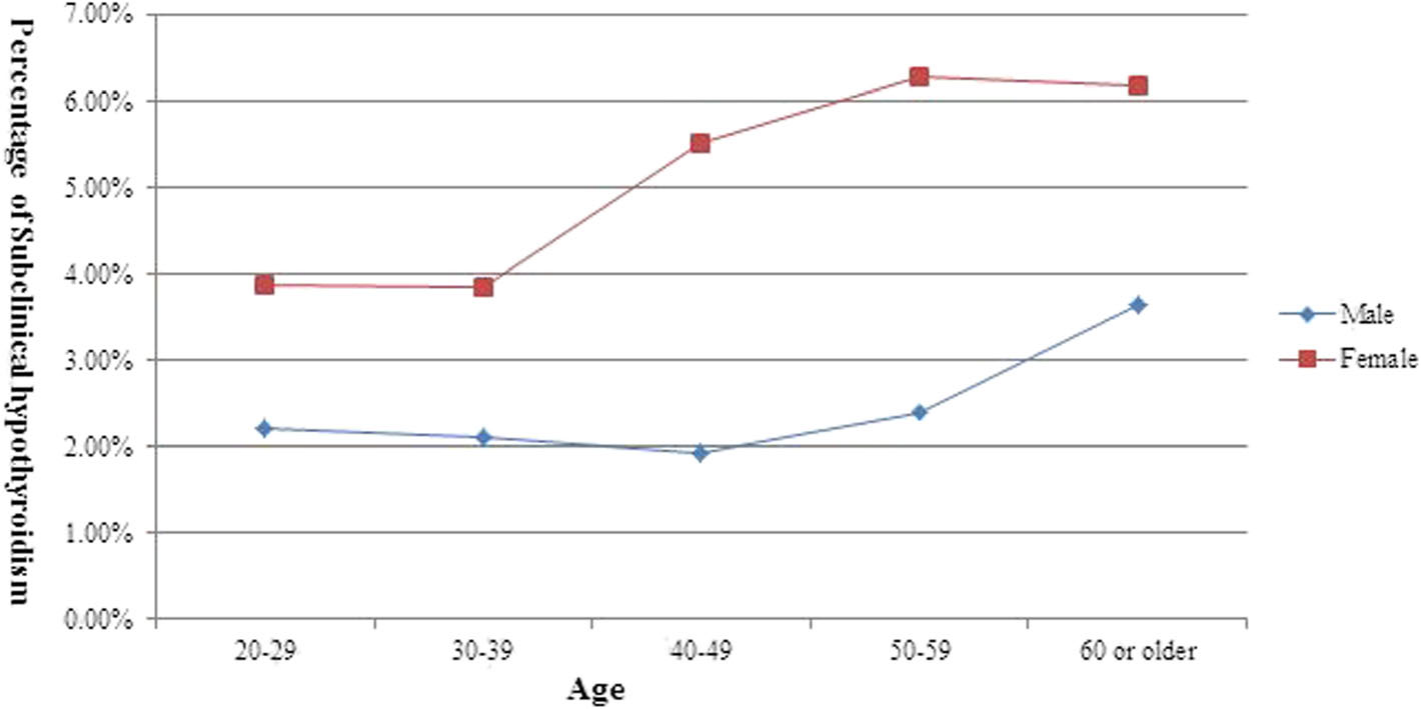
The age-specific prevalence curve of subclinical hypothyroidism by sex

The age-specific prevalence curve was different between male and female participants. The prevalence in each age category was higher in female participants than that in male participants. Compared to male participants, the prevalence in female participants increased 1.68, 1.75, 3.59, 3.89 and 2.53% in the 20 to 29, 30 to 39, 40 to 49, 50 to 59 and 60 or older age categories, respectively; and the largest difference was observed in the 50–59 age group (Fig. 2).

Additionally, the prevalence was 2.08% (57/2739) and 3.64% (10/274) in male participants younger than 60 and aged 60 or older, respectively; and the difference was not significant (*p* = 0.104). For female participants, the prevalence of SCH was 3.86% (44/1139) and 5.91% (69/1167) in the participants younger than 40 and aged 40 or older, respectively; the difference was significant (*p* = 0.023).

### Metabolic risk factors associated with SCH

After adjustment for age and BMI, in male participants, univariate analysis found that TGs were associated with the prevalence of SCH (*p* = 0.021), while other metabolic risk factors, including SBP/DBP, AC, UA and HDL-C, were not; in female participants, univariate analysis revealed that TGs (OR = 2.172, 95% CI 1.308–3.607, *p* = 0.006), SBP/DBP(OR = 1.730, 95% CI 1.138–2.630, *p* = 0.014) and AC (OR = 2.593, 95% CI 1.479–4.546, *p* < 0.001) were associated with the prevalence of SCH, while other metabolic risk factors UA and HDL-C were not.

Multivariate logistic regression analysis demonstrated that none of the metabolic risk factors were significantly associated with the prevalence of SCH in male participants (Table 2). However, in female participants, age (OR = 0.568, 95% CI 0.389–0.831, *p* = 0.004), SBP/DBP (OR = 2.543, 95% CI 1.709–3.784, *p* < 0.001), and BMI (OR = 5.029, 95% CI 3.306–7.6511, *p* < 0.001) were independent predictors of SCH (Table 3). Further analysis showed that there was an obvious interaction between age and SBP/DBP (OR = 4.027, 95% CI 1.604–10.110), but no significant interaction between age and BMI (OR = 1.024, 95% CI 0.462–2.271), or between SBP/DBP and BMI (OR = 1.4.6, 95% CI 0.640–3.091) (Table 3).

**Table 2:** Multivariate logistic regression analysis for predicting prevalence of SCH in male participants (*n* = 3013)

Variable	Hazard ratio	95% confidence interval		
		Lower	Upper	*p* value
Age	0.910	0.324	2.555	0.858
BMI	0.775	0.375	1.588	0.486
TG	0.735	0.237	2.279	0.447
Age by BMI ^**a**^	1.395	0.483	4.029	0.875
Age by TG ^**a**^	1.108	0.176	6.975	0.328
BMI by TG ^**a**^	2.260	0.509	10.035	0.314

^**a**^Interaction *p* value of variables for SCH

*Abbreviation*: *SCH* subclinical hypothyroidism, *BMI* body mass index, *TG* triglyceride

**Table 3:** Multivariate logistic regression analysis for predicting prevalence of SCH in female participants (*n* = 2306)

Parameter	Hazard ratio	95% confidence interval		
		Lower	Upper	*p* value
Age	0.568	0.389	0.831	0.004
TG	0.787	0.482	1.285	0.339
AC	1.329	0.871	2.028	0.187
SBP / DBP	2.543	1.709	3.784	0.000
BMI	5.029	3.306	7.651	0.000
Age by SBP /DBP ^**a**^	4.027	1.604	10.110	0.003
Age by BMI ^**a**^	1.024	0.462	2.271	0.954
BMI by SBP /DBP ^**a**^	1.406	0.640	3.091	0.396

^**a**^Interaction *p* value of every two predictors for SCH

*Abbreviation*: *SCH* subclinical hypothyroidism, *TG* triglyceride, *AC* fasting blood glucose, *SBP* systolic blood pressure, *DBP* diastolic blood pressure, *BMI* body mass index

### Prevalence of SCH according to metabolic risk factors

Based on two independent metabolic risk factors: SBP/DBP and BMI, we further classified the female participants into two subgroups: the low BMI and low blood pressure (BP) group, and the high BMI and/or high BP group. The prevalence of SCH differed significantly among the groups, regardless of age (Table 4).

**Table 4:** Prevalence of SCH with some metabolic risk factors in female participants (*n* = 2306)

Metabolic risk factors	Age < 40			Age ≥ 40		
	SCH (n (%))	EUT (n (%))	*p*	SCH (n (%))	EUT (n (%))	*p*
Low BP and low BMI	31 (3.26)	921 (96.74)	< 0.001	21 (3.09)	658 (96.91)	< 0.001
High BP and/or high BMI	18 (9.62)	169 (90.38)		43 (8.81)	445 (91.19)	

Data is presented as n

Low BP means systolic blood pressure ≤ 130 mmHg and diastolic blood pressure ≤ 85 mmHg; high BP means systolic blood pressure > 130 mmHg and/or diastolic blood pressure > 85 mmHg; low BMI means ≤25 kg/m2; high BMI means > 25 kg/m2

*Abbreviations*: *SCH* subclinical hypothyroidism, *EUT* euthyroid, *BP* blood pressure, *BMI* body-mass index

*P* < 0.05 is considered as statistically significance

## Discussion

This cross-sectional study is one of few studies investigating the sex differences in SCH in a large-scale health examination-based Chinese population. The current study revealed three major differences between male and female participants. First, the prevalence of SCH among female participants (4.90% in this study) was much higher than that among male participants (2.26% in this study), which is comparable with the results reported in some other studies [7–9], yet not all studies [22]. Second, the age-specific prevalence of SCH among female participants was consistently higher than that among males, and the largest difference was observed in 40–59 age group. Third, age, BP and BMI were associated with the prevalence of SCH among female participants. However, neither of metabolic risk factor was found to be associated with SCH in male participants, which is inconsistent with the previous studies in the general population [23–26].

MetS is a cluster of three or more of the following metabolic risk factors: obesity, hypertension, atherogenisis, hyperlipidaemia and hyperglycaemia. It is important to note that numerous cross-sectional studies have found that MetS and its components were related to SCH [27–30].

However, whether each of the components was related to SCH in Chinese population was still unknown. In our study, we focused on a special population, health examination-based population, who previously had no clear history of metabolic disease and medication treatment, and found that the prevalence of MetS was lower than that in previous literature [20, 25], because when the participants who had a history of metabolic disease were excluded from our study, the prevalence would certainly decrease. Additionally, as you all know, the prevalence of Mets increased as age older, however, in our study the rate of participants older than 60 was lower than 10% (468/5319). Therefore, compared to value of MetS, in the present study, the value of components may be much higher. Among 2306 female participants, 19.14% (672/2306) had high BP (systolic BP > 130 mmHg and/or diastolic BP > 85 mmHg), and 14.01% (323/2306) had high BMI (> 25 kg/m^2^), and 4.47% (103/2306) had both high BP and high BMI, which added up to nearly 30% (19.14% + 14.01%–4.47%) of participants who were at higher risk for SCH. In our study, as many as 9.04% of female participants who had at least one above metabolic risk factor were finally diagnosed with SCH, which suggested that TSH screening may be a suitable option for women with high BP and/or high BMI.

In previous literature, age and BMI were always included as adjustment factors when estimating SCH prevalence, and we also adopted the same statistical method in our analysis. After adjusting for age and BMI, the univariate analysis revealed that a high TG concentration was significantly associated with a high prevalence of SCH in male participants; however, the TG level was no longer an independent predictor of SCH after performing the multivariate logistic regression analysis. Similarly, the TG level was not an independent predictor of SCH in female participants. However, in female participants, we found three independent predictors, including age, BMI and BP. There was a 5.029-fold higher risk of SCH in female participants with high BMI (> 25 kg/m^2^) than in female participants with low BMI (≤ 25 kg/m^2^), and a 2.543-fold higher risk of SCH in female participants with high SBP/DBP (systolic BP > 130 mmHg and/or diastolic BP > 85 mmHg) than in female participants with low SBP/DBP (systolic BP ≤ 130 mmHg and diastolic BP ≤ 85 mmHg).

Regardless of age, the prevalence of SCH was much higher in the female participants with either or both high BP and high BMI than in participants with neither of these two factors. Although the prevalence of 9.04% (61/675) is not high, we believe that FT4 and TSH screening in this population has values. On the one hand, through routine TSH screening, it is possible to select about 9% of women with SCH, and these women may eventually develop to overt hypothyroidism each year at a rate of 4.3–8.0% [13, 31]. On the other hand, SCH is mainly caused by autoimmune diseases, such as Hashimoto’s thyroiditis, and levothyroxine replacement is the main treatment for SCH, which is appropriate according to current guidelines. Most importantly, several studies have revealed that SCH is correlated with an increased prevalence of coronary heart disease (CHD) or ischaemic heart disease [1, 4, 32–36], and CHD mortality in those with higher TSH levels, particularly in those with a TSH concentration of 10 μIU/mL or greater [36]. Moreover, a cochrane systematic review including 12 randomized controlled trials (RCTs) with a total of 350 patients showed that there was some evidence that levothyroxine replacement improved cardiac function and blood lipids, but a lack of data for improved survival, reduced cardiovascular morbidity or improved health-related quality of life [37]. However, the RCTs included in this systemic review were conducted before 2006 and the sample size of 350 was too small. An ongoing clinical trial, Thyroid hormone Replacement for Untreated older adults with Subclinical hypothyroidism - a randomised placebo-controlled Trial (TRUST) will answer the unsettled questions: whether levothyroxine replacement can change the symptoms related to SCH; whether levothyroxine replacement has impact on metabolic risk factors; and whether levothyroxine replacement has any benefit for decreasing incident atrial fibrillation, heart failure and bone fracture [38].

The strengths of this study should be acknowledged. First, a large number of participants were assessed retrospectively, and all participants underwent serum FT4 and TSH tests and metabolic risk factor measurements within a relatively short interval so that detection bias could be minimized. Second, the upper limit of normal TSH concentration adopted in the current study was 5.0μIU/mL, which is widely used clinically in mainland China. Third and most importantly, in our study, age, BMI and BP, which were found to be the predictors of SCH in female participants, were easily determined before blood samples were taken, which indicates that it is reasonable that the estimated risk for SCH could be calculated, and the decision whether the TSH test was necessary could be easily made.

Of course, there are some limitations in this study. First, although high BP and high BMI were confirmed as independent predictive factors for SCH, our cross-sectional study could not determine the longitudinal effects of SCH, the causation of SCH, or the metabolic risk factors or the underlying mechanism could not be explored. Second, the predictive values of factors such as area of residence, iodine intake and the presence of autoimmune antibodies, which have been associated with SCH in previous literature [8–10], could not be evaluated in this study. Third, the participants whose TSH concentration was higher than 10 mIU/L were excluded from our study; thus, we could not further explore the associations between metabolic risk factors and SCH in those patients. However, it is most likely that there was much stronger association of SCH with TSH > 10 mU/l than TSH < 10 mU/l and metabolic factors, and in the near future we are going to conduct a a prospective cohort study to further explore the metabolic factors and SCH with TSH > 10 mU/l.

## Conclusions

Our study shows that in female participants, high BMI and high BP are associated with SCH. Considering that female participants with either or both of above metabolic risk factors have a prevalence of 7.69–14.81% for SCH, together with a continuous increase in the rate of SCH [23], serum concentrations of TSH and FT4 in such populations may be routinely monitored. Of course, a prospective, large-scale study with long follow-up period is still needed to verify our results.

## Data Availability

The datasets generated during and analyzed during the current study are available from the corresponding author on reasonable request.

## Abbreviations

SCH: Subclinical hypothyroidism
TSH: Thyroid-stimulating hormone
MS: Metabolic syndrome
BMI: Body-mass index
AC: Fasting blood glucose
HDL-C: High-density lipoprotein cholesterol
LDL-C: Low-density lipoprotein cholesterol
TG: Triglyceride
UA: Uric acid
Scr: Serum creatinine
FT4: Free tetraiodothyronine
FT3: Free triiodothyronine
EUT: Euthyroid
OR: Odds ratio
CI: Confidence interval
BP: Blood pressure

## References

[1] Rodondi N, den Elzen WP, Bauer DC, Cappola AR, Razvi S, Walsh JP, Asvold BO, Iervasi G, Imaizumi M, Collet TH, Bremner A, Maisonneuve P, Sgarbi JA, Khaw KT, Vanderpump MP, Newman AB, Cornuz J, Franklyn JA, Westendorp RG, Vittinghoff E, Gussekloo J (2010). Thyroid studies collaboration. Subclinical hypothyroidism and the risk of coronary heart disease and mortality. JAMA..

[2] Danese MD, Ladenson PW, Meinert CL, Powe NR (2000). Clinical review 115: effect of thyroxine therapy on serum lipoproteinsin patients with mild thyroid failure: a quantitative review of the literature. J Clin Endocrinol Metab.

[3] Haggerty JJ, Stern RA, Mason GA, Beckwith J, Morey CE, Prange AJ (1993). Subclinical hypothyroidism: a modifiable risk factor for depression?. Am J Psychiatry.

[4] Tseng FY, Lin WY, Lin CC, Lee LT, Li TC, Sung PK, Huang KC (2012). Subclinical hypothyroidism is associated with increased risk for all-cause and cardiovascular mortality in adults. J Am Coll Cardiol.

[5] Tunbridge WM, Evered DC, Hall R, Appleton D, Brewis M, Clark F, Grimley Evans J, Young E, Bird T, Smith PA (1997). The spectrum of thyroid disease in a community: the Whickham survey. Clin Endocrinol.

[6] Canaris GJ, Manowitz NR, Mayor G, Ridgway EC (2000). The Colorado thyroid disease prevalence study. Arch Intern Med.

[7] Razvi S, Weaver JU, Pearce SH (2010). Subclinical thyroid disorders: significance and clinical impact. J ClinPathol.

[8] Hollowell JG, Staehling NW, Dana Flanders W, Harry Hannon W, Gunter EW, Spencer CA, Braverman LE (2002). Serum TSH, T (4), and thyroid antibodies in the United States population (1988 to 1994): National Health and nutrition examination survey (NHANES III). J Clin Endocrinol Metab.

[9] Kim WG, Kim WB, Woo G, Kim H, Cho Y, Kim TY, Kim SW, Shin MH, Park JW, Park HL, Oh K, Chung JH (2017). Thyroid stimulating hormone reference range and prevalence of thyroid dysfunction in the Korean population: Korea National Health and nutrition examination survey 2013 to 2015. Endocrinol Metab (Seoul).

[10] Laurberg P, Pedersen KM, Hreidarsson A, Sigfusson N, Iversen E, Preben R, Knudsen PR (1998). Iodine intake and the pattern of thyroid disorders: a comparative epidemiological study of thyroid abnormalities in the elderly in Iceland and in Jutland, Denmark. J Clin Endocrinol Metab.

[11] Surks MI, Ortiz E, Daniels GH (2004). Subclinical thyroid disease: scientific review and guidelines for diagnosis and management. JAMA..

[12] Fliers E, Bianco AC, Langouche L, Boelen A (2015). Thyroid function in critically ill patients. Lancet Diabetes Endocrinol.

[13] Baskin HJ, Cobin RH, Duick DS, Gharib H, Guttler RB, Kaplan MM, Segal RL, Garber JR, Hamilton CR, Handelsman Y, Hellman R, Kukora JS, Levy P, Palumbo PJ, Petak SM, Rettinger HI, Rodbard HW, Shankar TP, Stoffer SS, Tourtelot JB, Service FJ (2002). American Association of Clinical Endocrinologists medical guidelines for clinical practice for the evaluation and treatment of hyperthyroidism and hypothyroidism. Endocr Pract.

[14] Jonklaas J, Bianco AC, Bauer AJ, Burman KD, Cappola AR, Celi FS, Cooper DS, Kim BW, Peeters RP, Rosenthal MS, Sawka AM (2014). American Thyroid Association task force on thyroid hormone replacement. Guidelines for the treatment of hypothyroidism: prepared by the American Thyroid Association task force on thyroid hormone replacement. Thyroid..

[15] Garber JR, Cobin RH, Gharib H, Hennessey JV, Klein I, Mechanick JI, Pessah-Pollack R, Singer PA, Woeber KA (2012). Clinical practice guidelines for hypothyroidism in adults: cosponsored by the American Association of Clinical Endocrinologists and the American Thyroid Association. Endocr Pract.

[16] Leroith D (2012). Pathophysiology of the metabolic syndrome: implications for the cardiometabolic risks associated with type 2 diabetes. Am J Med Sci.

[17] Lai Y, Wang J, Jiang F, Wang B, Chen Y, Li M, Liu H, Li C, Xue H, Li N, Yu J, Shi L, Bai X, Hou X, Zhu L, Lu L, Wang S, Xing Q, Teng X, Teng W, Shan Z (2011). The relationship between serum thyrotropin and components of metabolic syndrome. Endocr J.

[18] Bonora BM, Fadini GP (2016). Subclinical hypothyroidism and metabolic syndrome: a common association by chance or a cardiovascular risk driver?. Metab Syndr Relat Disord.

[19] Liu C, Scherbaum WA, Schott M, Schinner S (2011). Subclinical hypothyroidism and the prevalence of the metabolic syndrome. Horm Metab Res.

[20] Chang CH, Yeh YC, Caffrey JL, Shih SR, Chuang LM, Tu YK (2017). Metabolic syndrome is associated with an increased incidence of subclinical hypothyroidism–a cohort study. Sci Rep.

[21] Cheserek MJ, Wu G, Shen L, Shi Y, Le G (2014). Evaluation of the relationship between subclinical hypothyroidism and metabolic syndromecomponents among workers. Int J Occup Med Environ Health.

[22] Legakis I, Manousaki M, Detsi S, Nikita D (2013). Thyroid function and prevalence of anti-thyroperoxidase (TPO) and anti-thyroglobulin (Tg) antibodies in outpatients hospital setting in an area with sufficient iodine intake: influences of age and sex. Acta Med Iran.

[23] Chang YC, Chang CH, Yeh YC, Chuang LM, Tu YK (2018). Subclinical and overt hypothyroidism is associated with reduced glomerular filtration prevalence and proteinuria: a large cross-sectional population study. Sci Rep.

[24] Pesic MM, Radojkovic D, Antic S, Kocic R, Stankovic-Djordjevic D (2015). Subclinical hypothyroidism: association with cardiovascular risk factors and components of metabolic syndrome. Biotechnol Biotechnol Equip.

[25] Liu FH, Hwang JS, Kuo CF, Ko YS, Chen ST, Lin JD (2018). Subclinical hypothyroidism and metabolic risk factors association: a health examination-based study in northern Taiwan. Biom J.

[26] Khatiwada S, Sah SK, Kc R, Baral N, Lamsal M (2016). Thyroid dysfunction in metabolic syndrome patients and its relationship with components of metabolic syndrome. Clin Diabetes Endocrinol.

[27] Knudsen N, Laurberg P, Rasmussen LB, Bülow I, Perrild H, Ovesen L, Jørgensen T (2005). Small differences in thyroid function may be important for body mass index and the occurrence of obesity in the population. JCEM..

[28] Roos A, Bakker SJ, Links TP, Gans RO, Wolffenbuttel BH (2007). Thyroid function is associated with components of the metabolic syndrome in euthyroid subjects. J Clin Endocrinol Metab.

[29] Ruhla S, Weickert MO, Arafat AM, Osterhoff M, Isken F, Spranger J, Schöfl C, Pfeiffer AF, Möhlig M (2010). A high normal TSH is associated with the metabolic syndrome. Clin Endocrinol.

[30] Nader NS, Bahn RS, Johnson MD, Weaver AL, Singh R, Kuma S (2010). Relationships between thyroid function and lipid status or insulin resistance in a pediatric population. Thyroid..

[31] Szabolcs I, Podoba J, Feldkamp J, Dohan O, Farkas I, Sajgó M, Takáts KI, Góth M, Kovács L, Kressinszky K, Hnilica P, Szilágyi G, Szabolcs I, Podoba J, Feldkamp J (1997). Comparative screening for thyroid disorders in old age in areas of iodine deficiency, long term iodine prophylaxis and abundant iodine intake. Clin Endocrinol.

[32] Biondi B, Klein I (2004). Hypothyroidism as a risk factor for cardiovascular disease. Endocrine..

[33] Duntas LH, Wartofsky L (2007). Cardiovascular risk and subclinical hypothyroidism: focus on lipids and new emerging risk factors. What is the evidence?. Thyroid..

[34] Biondi B, Cooper DS (2008). The clinical significance of subclinical thyroid dysfunction. Endocr Rev.

[35] Walsh JP, Bremner AP, Bulsara MK, O'Leary P, Leedman PJ, Feddema P, Michelangeli V (2005). Subclinical thyroid dysfunction as a risk factor for cardiovascular disease. Arch Intern Med.

[36] Collet TH, Bauer DC, Cappola AR, Asvold BO, Weiler S, Vittinghoff E, Gussekloo J, Bremner A, den Elzen WP, Maciel RM, Vanderpump MP, Cornuz J, Dörr M, Wallaschofski H, Newman AB, Sgarbi JA, Razvi S, Völzke H, Walsh JP, Aujesky D, Rodondi N (2014). Thyroid studies collaboration. Thyroid antibody status, subclinical hypothyroidism, and the risk of coronary heart disease: an individual participant data analysis. J Clin Endocrinol Metab.

[37] Villar HC, Saconato H, Valente O, Atallah AN (2007). Thyroid hormone replacement for subclinical hypothyroidism. Cochrane Database Syst Rev.

[38] Stott DJ, Gussekloo J, Kearney PM, Rodondi N, Westendorp RG, Mooijaart S, Kean S, Quinn TJ, Sattar N, Hendry K, Du Puy R, Den Elzen WP, Poortvliet RK, Smit JW, Jukema JW, Dekkers OM, Blum M, Collet TH, McCarthy V, Hurley C, Byrne S, Browne J, Watt T, Bauer D, Ford I (2017). Study protocol; Thyroid hormone Replacement for Untreated older adults with Subclinical hypothyroidism - a randomised placebo controlled Trial (TRUST). BMC Endocr Disord.

